# Supplementary material for: Dopamine-Mediated Graphene Bridging Hexagonal Boron Nitride for Large-Scale Composite Films with Enhanced Thermal Conductivity and Electrical Insulation
Source: Nanomaterials (Basel). 2023 Mar 29;13(7):1210. doi: 10.3390/nano13071210 (PMC10097086; doi:10.3390/nano13071210)
Supplement: Supplementary file 1 [file nanomaterials-13-01210-s001.zip › nanomaterials-2261575-supplementary.pdf]

nanomaterials

Supplementary Materials

Dopamine-Mediated Graphene Bridging Hexagonal Boron Nitride for Large-Scale Composite Films with Enhanced Thermal Conductivity and Electrical Insulation

Shikun Li [1,2,3], Yutan Shen [4], Xiao Jia [2,3], Min Xu [2,3], Ruoyu Zong [2,3], Guohua Liu [1], Bin Liu [2,3,*] and Xiulan Huai [2,3,*]

1 Beijing Key Laboratory of Multiphase Flow and Heat Transfer for Low Grade Energy Utilization, North China Electric Power University, Beijing 102206, China
2 Institute of Engineering Thermophysics, Chinese Academy of Sciences, Beijing 100190, China
3 Nanjing Institute of Future Energy System, Nanjing 211135, China
4 SINOPEC Research Institute of Petroleum Processing Co., Ltd., Beijing 100083, China
* Correspondence: hxl@iet.cn (X.H.); liubin@iet.cn (B.L.)

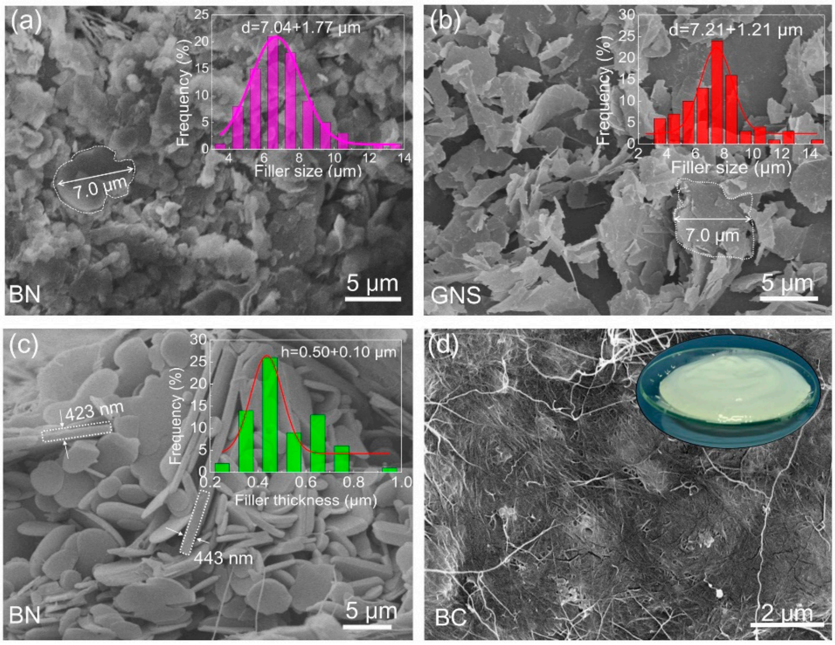

Figure S1. (a,b) SEM images and filler size distributions of BN and GNS fillers. (c) The measered thicknesses of the BN filler, which are indicated by white lines and arrows. (d) SEM images of BC nanofibers, and the inset shows an optical photo of the BC solution.

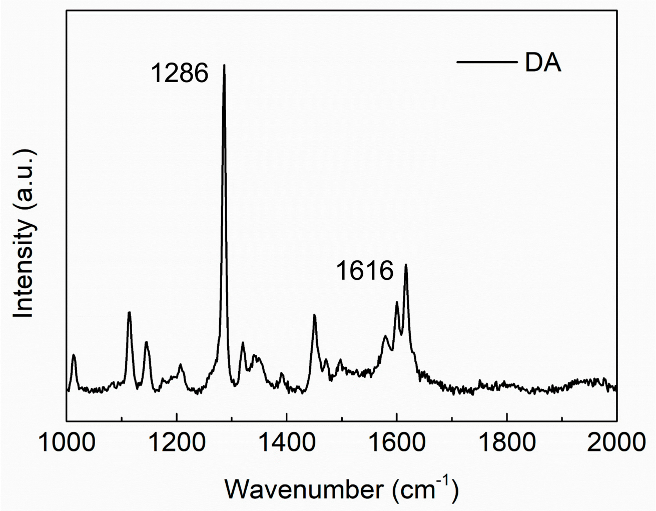

Figure S2. Raman spectra of DA.

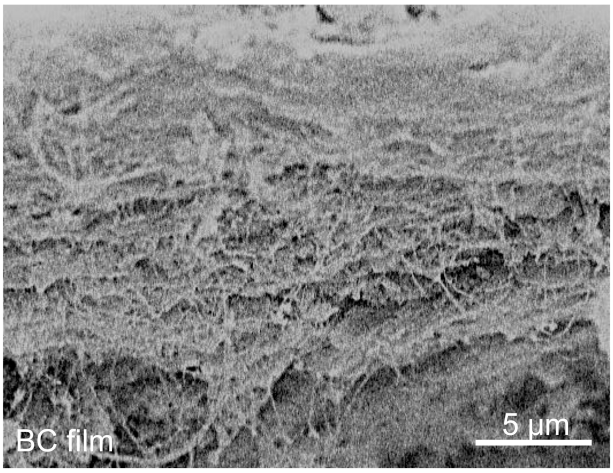

Figure S3. SEM images of the cross-section of a BC composite film.

S1. Calculation of thermal conductivity.

Table S1. The in-plane ($\alpha_{\parallel}$) and through-plane ($\alpha_{\perp}$) thermal diffusivity, density (ρ), and specific heat capacity (C) of composite films at different filler contents.

Sample	$\alpha_{\parallel}$ (mm^2·s^{-1})	$\alpha_{\perp}$ (mm^2·s^{-1})	ρ (g·cm^{-3})	C (J·g^{-1}·K^{-1})
BC	2.46	0.206	1.3	0.94
BN	–	–	2.2	0.91
GNS	–	–	2.2	1.40
10 wt % BC/BN_{PDA}	3.454	0.228	1.390	0.937
30 wt % BC/BN_{PDA}	7.194	0.269	1.597	0.930
50 wt % BC/BN_{PDA}	10.127	0.312	1.750	0.925
70 wt % BC/BN_{PDA}	12.221	0.408	1.930	0.919
10 wt % BC/BN_{PDA}/GNS_{PDA}-5	3.744	0.209	1.390	0.941
30 wt % BC/BN_{PDA}/GNS_{PDA}-5	8.415	0.231	1.597	0.933
50 wt % BC/BN_{PDA}/GNS_{PDA}-5	13.465	0.254	1.750	0.929
70 wt % BC/BN_{PDA}/GNS_{PDA}-5	19.657	0.274	1.930	0.923
70 wt % BC/BN_{PDA}/GNS_{PDA}-1	12.221	–	1.930	0.919
70 wt % BC/BN_{PDA}/GNS_{PDA}-3	13.721	–	1.930	0.921
70 wt % BC/BN_{PDA}/GNS_{PDA}-7	20.486	–	1.930	0.924
70 wt % BC/BN_{PDA}/GNS_{PDA}-10	21.359	–	1.930	0.927

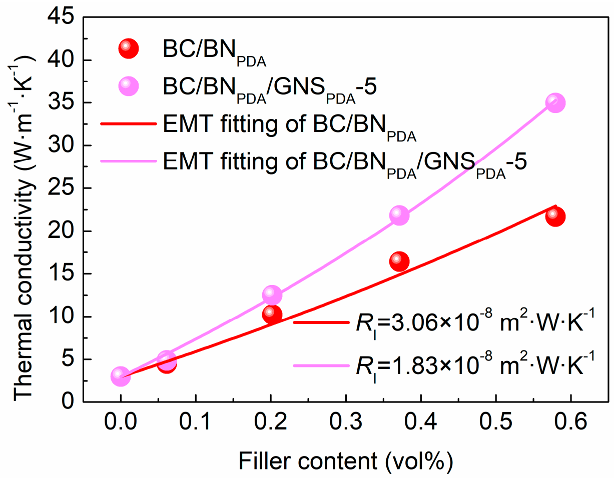

Figure S4. The R_l values were calculated by fitting the in-plane thermal conductivities of the BC/BN$_{PDA}$ and BC/BN$_{PDA}$/GNS$_{PDA}$-5 composite film based on the EMT model.

S2. The calculation of thermal contact resistance.

When phonons propagate through adjacent hybrid fillers in the films, the thermal contact resistance between the connected "BN-GNS-BN" pathways dominates heat transfer efficiency of the films. Such behavior of phonon transport between adjoining hybrid fillers can be theoretically explained by Foygel theory,[1] which is defined as the following equation:

$$k - k_{p} = k_{0}\left[\frac{f - f_{c}}{1 - f_{c}}\right]^{\tau} \quad \text{(S1)}$$

where k and k_p are the thermal conductivities of the composite films and the BC matrix, respectively; k_0 is a pre-exponential factor ratio caused by interconnected hybrid fillers; f is the measured volume fraction of hybrid filler in the films; τ is the conductivity exponent determined by the aspect ratio of BN; and f_c is the critical volume fraction of the hybrid fillers, which is the intercept of the tangent with the x-axis in the figure of thermal conductivity of the composites as a function of filler content. In our case, f_c in the case of BC/BN$_{PDA}$ and BC/BN$_{PDA}$/GNS$_{PDA}$-5 is 10 vol% and 15 vol%, respectively, as shown in Figure S3.

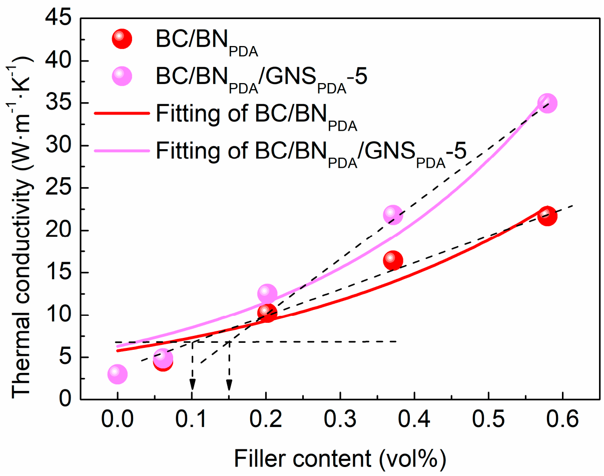

Figure S5. Foygel model fitting curves of BC/BN$_{PDA}$ and BC/BN$_{PDA}$/GNS$_{PDA}$-5 composite films, respectively.

In order to obtain the values of k_0 and τ, Equation S1 needs to be converted into a linear form:

$$\lg\left(k-k_{\mathrm{p}}\right)=\lg k_0+\tau\lg\left(\frac{f-f_{\mathrm{c}}}{1-f_{\mathrm{c}}}\right) \tag{S2}$$

Equation S2 is can be seen as the linear equation of $y = ax + b$. Therefore, the values of k_0 and τ can be calculated by linear fitting from our experiments, as shown in Figure S4.

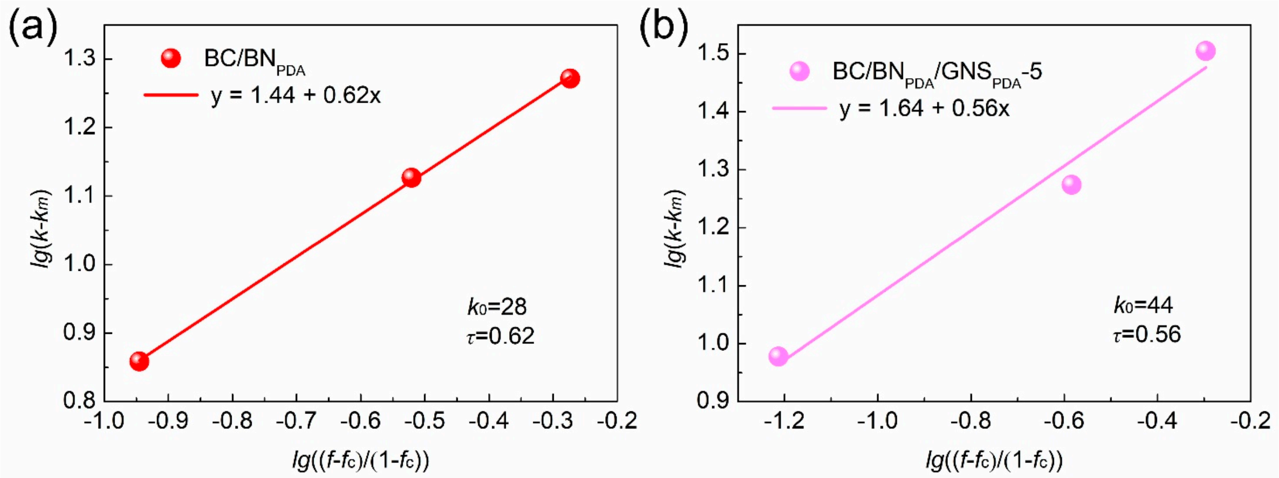

Figure S6. Linear fitting results based on Foygel model for (a) BC/BN$_{PDA}$ and (b) BC/BN$_{PDA}$/GNS$_{PDA}$-5 composite films.

Based on the calculated values of k_0 and τ, we can obtain the thermal contact resistance (R_c) between the adjacent hybrid filler using the following equation:

$$R_{\mathrm{c}}=\frac{1}{k_0 L V_{\mathrm{c}}^{\tau}} \tag{S3}$$

Where L is the lateral size of main h-BN filler, which is 7.0 μm (Figure S1). As a result, the calculated R_c of BC/BN$_{PDA}$ composite film is 2.1 × 10^5 K·W^{-1}, which is approximately two times higher than that of BC/BN$_{PDA}$/GNS$_{PDA}$-5 (0.9 × 10^5 K·W^{-1}).

S3. Simulation of the finite element models, the grid divisions, and the calculation method.

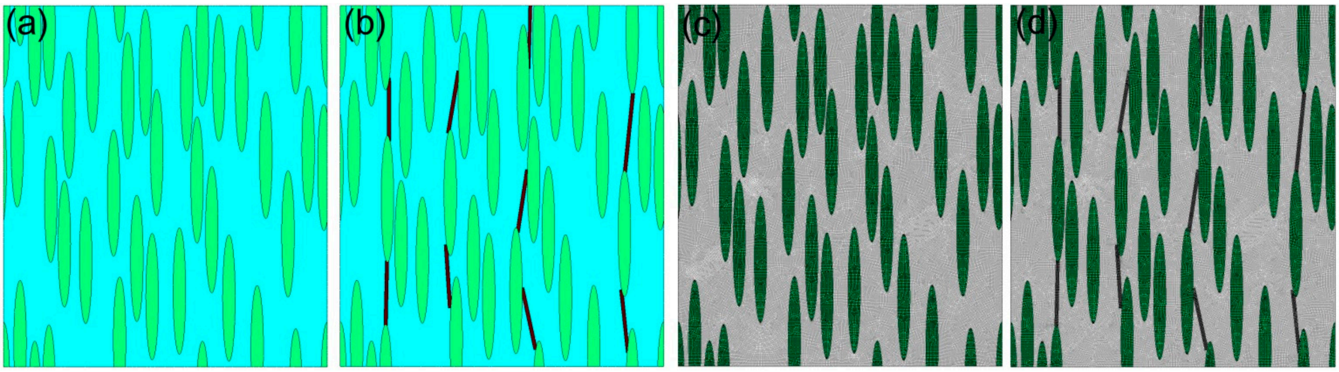

Figure S7. (a,b) The finite element models of the composite films: BC/BN$_{PDA}$ and BC/BN$_{PDA}$/GNS$_{PDA}$-5. (c,d) The mesh distributions of the composite films: BC/BN$_{PDA}$ and BC/BN$_{PDA}$/GNS$_{PDA}$-5.

The finite element simulation software (ANSYS) was employed to investigate the small amounts of flexible GNS filler (5 wt %) on the heat transfer performance of the composite films. The values of 323 and 293 K were set at the left and right boundaries of the finite element models. The length and width of the finite element model were 9 and 12 μm, respectively, and the volume fraction of hybrid fillers was 50%. The BN was simplified to an ideal oval, and the major and minor axes were 7.0 and 0.5 μm. Similarly, the GNS filler was simplified to a rectangle, and the length and width were 7.0 and 0.2 μm. The thermal conductivity of BN was set to 180 $W \cdot m^{-1} \cdot K^{-1}$, as seen in the literature.[2] The thermal conductivity of GNS was set to 600 $W \cdot m^{-1} \cdot K^{-1}$, as seen in the literature,[3] and the thermal conductivity of the BC matrix was set to 3.05 $W \cdot m^{-1} \cdot K^{-1}$, as measured in Table S1. The quadrilateral element was used to mesh the mode, as shown in Figure S5c–d.

The thermal conductivity of finite element models was obtained according to the Fourier law:[4]

$$k = \frac{ql}{\Delta T} \tag{S4}$$

where k is the effective thermal conductivity, q is the heat flow rate, ΔT is the temperature difference between left and right boundary, and l is the length of the finite element model. The heat flow rate, q, across the left and right boundary was calculated by integrating the heat flux to the corresponding boundary.

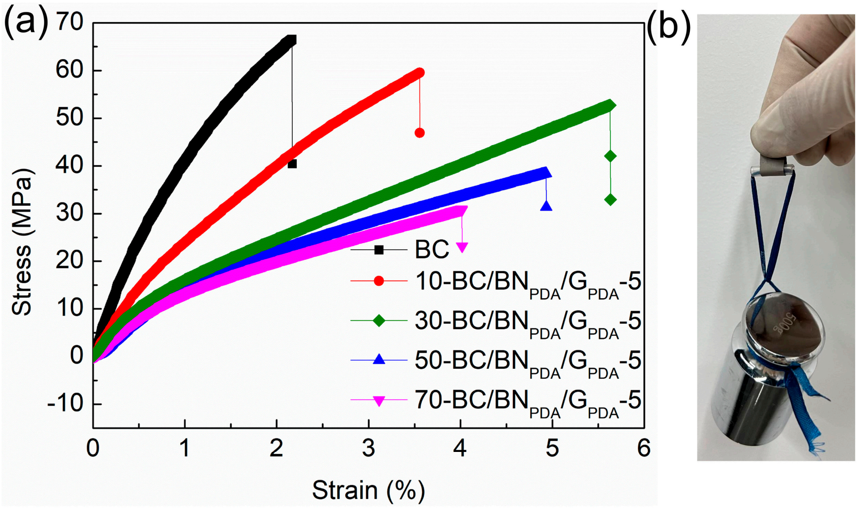

Figure S8. (a) Stress–strain curves of BC/BNPDA/GNSPDA-5 composite films at varied filler content. (b) The optical image of the 70 wt % BC/BNPDA/GNSPDA-5 film lifting a 500 g weight.

References

1. Shi, X.; Wang, K.; Tian, J.; Yin, X.; Guo, B.; Xi, G.; Wang, W.; Wu, W. Few-Layer Hydroxyl-Functionalized Boron Nitride Nano sheets for Nanoscale Thermal Management. *ACS Appl. Nano Mater.* **2020**, *3*, 2310–2321.
2. Li, S.; Liu, B.; Jia, X.; Xu, M.; Liu, Z.; Liu, G.; Huai, X. Dopamine-Mediated Bacterial Cellulose/Hexagonal Boron Nitride Composite Films with Enhanced Thermal and Mechanical Performance. *Ind. Eng. Chem. Res.* **2022**, *61*, 4601–4611.
3. Wang, T.; Tsai, J. Investigating thermal conductivities of functionalized graphene and graphene/epoxy nanocomposites. *Comput. Mater. Sci.* **2016**, *122*, 272-280.
4. Tong, Z.; Liu, M.; Bao, H. A numerical investigation on the heat conduction in high filler loading particulate composites. *Int. J. Heat Mass Transf.* **2016**, *100*, 355-361.
